# A novel prognostic prediction indicator in patients with acute pulmonary embolism: Naples prognostic score

**DOI:** 10.1186/s12959-023-00554-8

**Published:** 2023-11-06

**Authors:** Ning Zhu, Shanhong Lin, Chao Cao

**Affiliations:** 1 Department of Respiratory and Critical Care Medicine, Key Laboratory of Respiratory Disease of Ningbo, The First Affiliated Hospital of Ningbo University, 59 Liuting Road, Ningbo, 315010 Zhejiang China; 2 Department of Ultrasound, The First Affiliated Hospital of Ningbo University, Ningbo, China

**Keywords:** Acute Pulmonary Embolism, Naples Prognostic score, Mortality, Prediction

## Abstract

Acute pulmonary embolism (APE) is a potentially fatal disease. Early risk stratification is essential to determining appropriate treatment. We aimed to investigate the predictive value of the Naples Prognostic Score (NPS) for 30-day all-cause mortality in patients with APE. In this retrospective analysis, 325 hospitalized patients with APE were divided into Groups 0 (n = 131), 1 (n = 153), and 2 (n = 41) according to the NPS. The primary outcome event was all-cause mortality during 30 days of follow-up from the day of admission. The correlation between NPS, clinical features, and outcomes in each group was evaluated. The patients were divided into two groups, survivor (n = 294) and nonsurvivor (n = 31), according to their prognosis. The results of the comparison between the three NPS groups revealed that patients with older age, faster heart rate, lower systolic blood pressure, low albumin and total cholesterol levels, high neutrophil to lymphocyte ratio (NLR), low lymphocyte-to-monocyte ratio (LMR), right heart dilatation, heart failure, malignancy, and lower extremity venous thrombosis had significantly higher 30-day all-cause mortality (P < 0.05). Area under the receiver operating characteristic curve (AUC) for NPS to predict all-cause death within 30 days in patients with APE was 0.780 (95% confidence interval [CI] = 0.678–0.855), with sensitivity being 80.6% (95% CI = 0.667–0.946) and specificity being 72.1% (95% CI = 0.670–0.772). Kaplan-Meier (KM) curves showed that Group 2 APE patients had the highest risk of all-cause mortality compared with the other two groups (log-rank test, P = 0.0004). Forest plot visualization using the Cox proportional hazard model showed a significant increase in the risk of 30-day all-cause mortality by 239% (hazard ratio [HR] = 3.385 [1.115–10.273], P = 0.031) and 338% (HR = 4.377 [1.228–15.598], P = 0.023), and the trend test showed a statistical difference (P = 0.042). The study concluded that NPS is a novel, reliable, and multidimensional prognostic scoring system with good prediction of 30-day all-cause mortality in patients with APE.

## Introduction

Acute pulmonary embolism (APE) is a clinical syndrome with respiratory and circulatory dysfunction as the main pathophysiological features [1]. The incidence of APE is 0.4–1.0 per 1,000 people, and this condition is known to have high missed diagnosis and mortality rates [1]. Early risk stratification is essential to determining appropriate treatment. Currently, the Pulmonary Embolism Severity Index (PESI) score [2] is mostly used clinically to assess the risk of death in patients with pulmonary embolism. However, the complexity of risk stratification by PESI, the need for multiple examinations, the time it takes, the high cost, high risk of the tests, and the limitations of the hospital that prevent rapid assessment of the disease [3]. Consequently, studies have addressed this issue by deriving a simplified version of the PESI in which some variables of the original score would be removed and the scoring system would be simplified [4]. Therefore there is a need to explore a new, cost-effective tool for prognostic assessment of APE. Various inflammatory or nutrition-related markers, including serum cholesterol levels, neutrophil-to-lymphocyte ratio (NLR), and lymphocyte-to-monocyte ratio (LMR), were reported to be possibly associated with the prognosis of patients with pulmonary embolism [3–5]. However, most predictors involved in previous studies were single inflammatory or nutritional markers that provide limited information for clinicians and produce controversial results. Therefore, more consistent, comprehensive, and validated risk assessment algorithms are required for patients with pulmonary embolism.

The Naples Prognostic Score (NPS) is a new scoring system that shows a patient’s inflammatory and nutritional status based on albumin (Alb) levels, total cholesterol (TC) levels, LMR, and NLR. It was initially identified as an independent prognostic marker for surgical survival in certain cancers (e.g., endometrial and gastrointestinal cancers), and subsequent studies have reported that NPS has a higher prognostic predictive value than other inflammatory markers and scoring systems [5–7]. According to two recent studies, NPS is extremely useful in the prognosis and prediction of nonmalignant neoplasms [8, 9]. NPS is a comprehensive prognostic model that is easily accessible, simple, and reliable; however, its clinical significance and prognostic assessment have not been reported in patients with APE. In view of this, this study aimed to investigate the relationship between NPS and its short-term prognosis at the time of admission of patients with APE in order to provide a novel predictive tool to guide the clinical decision-making process and prognostic assessment of patients with APE.

## Materials and methods

### Patients and study design

**Fig. 1:**
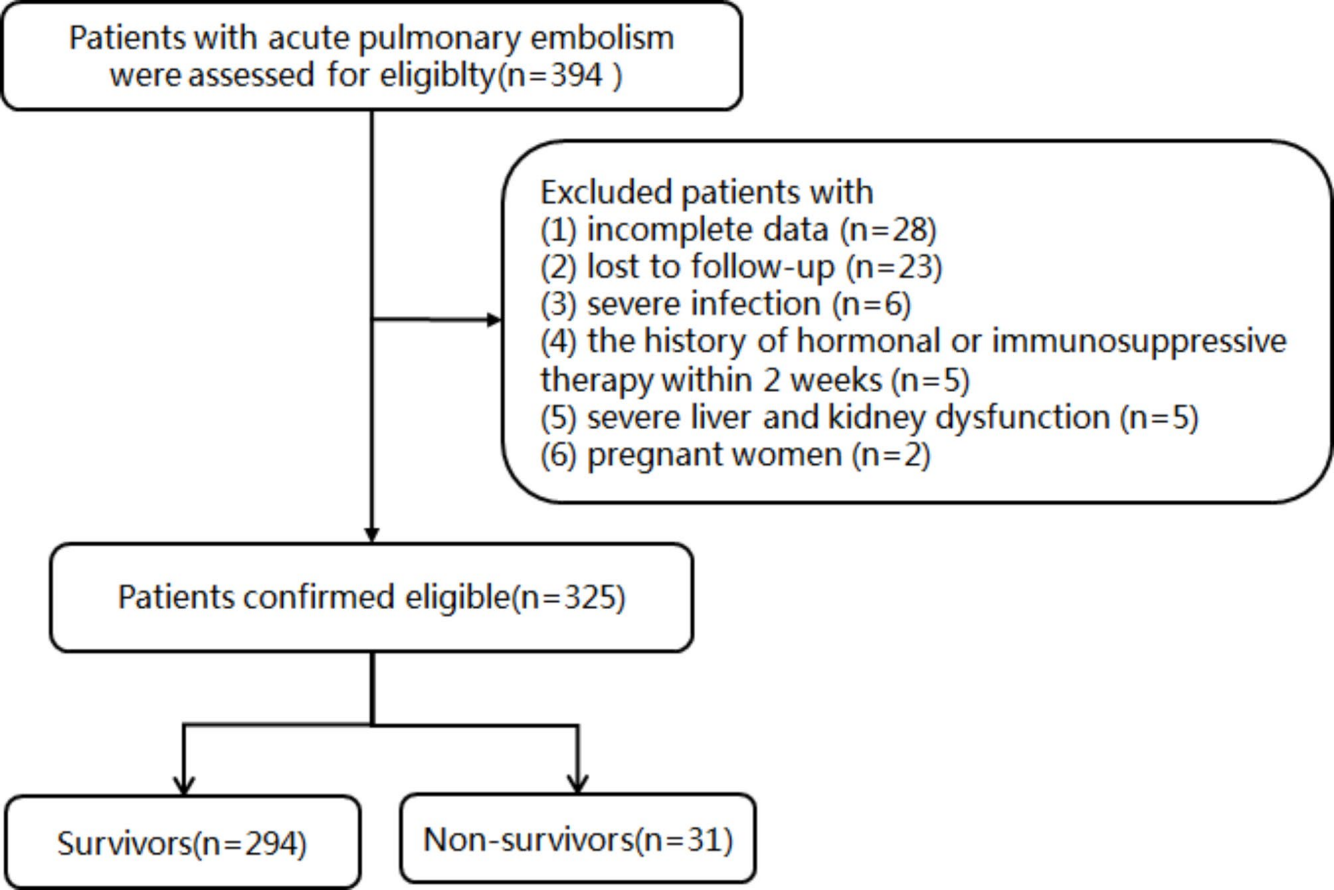
Flow diagram of the selected study population

Clinical data of patients diagnosed with APE who were admitted to the First Affiliated Hospital of Ningbo University from August 2015 to March 2023 was retrospectively collected. Inclusion criteria were as follows: (i) all patients with APE were diagnosed based on computer tomography pulmonary angiography (CTPA) with signs of hypointense filling defect of the pulmonary artery with distal vessel opacification by CTPA; (ii) complete information on inflammation and nutrition-related peripheral blood laboratory tests, including serum Alb levels, total cholesterol levels, NLR, and LMR. Exclusion criteria were as follows: (i) patients with a history of APE or chronic pulmonary embolism; (ii) pregnant and puerperal women; (iii) severe infection prior to admission; (iv) immunosuppressant or hormone use within 2 weeks; (v) severe liver and kidney dysfunction; (vi) transfusion of blood, Alb, and other blood products prior to admission; (vii) presence of other diseases causing abnormal serum Alb and cholesterol (e.g., nephrotic syndrome and active tuberculosis); and (viii) incomplete data or data lost to follow-up. Twenty-three patients were lost to follow-up. A total of 325 APE participants were enrolled in this study according to the inclusion and exclusion criteria. The primary outcome event was all-cause death at 30 days of follow-up from the day of admission. The 30-day mortality was identified via medical records or by contacting patients or their relatives regarding the status of survival or date of death. Patients who died within 30 days of hospital admission were defined as short-term death. Patients were classified as nonsurvivors and survivors according to their follow-up outcomes, and the flow chart of the study is shown in Fig. 1. This study was approved by the medical ethics committee of our hospital (No. 2023-047RS) and was conducted in accordance with the Declaration of Helsinki. The requirement for written informed consent was exempted because of its retrospective nature.

### Observation markers and detection methods

The following data of the included patients were collected: demographic data (age, sex, smoking history, body mass index [BMI], respiratory rate, heart rate, and systolic blood pressure); history of previous diseases (comorbid diabetes, hypertension, heart failure, chronic obstructive pulmonary disease, cerebrovascular disease, malignancy, and deep vein thrombosis); venous blood markers within 24 h of first admission (white blood cells, neutrophils, lymphocytes, monocytes, hemoglobin, platelets, total cholesterol, Alb, D-dimer, troponin, and NT-proBNP); arterial blood gas analysis markers (pH, lactate, partial pressure of oxygen[PaO_2_], and HCO_3_) within 24 h of first admission; CTPA and echocardiography within 72 h of admission; the location of thrombus in CTPA (pulmonary artery trunk, lobar pulmonary artery, segmental pulmonary artery, and subsegmental pulmonary artery); and echocardiography markers (right ventricular dilatation, left ventricular ejection fraction [LVEF], and pulmonary artery systolic pressure). NLR and LMR were calculated from the above corresponding markers, and the NPS was further calculated according to the scoring system [10]**(**Table 1). The patients were divided into the following three groups according to the NPS: patients with an NPS of 0 (n = 131) were defined as Group 0, patients with an NPS of 1–2 (n = 153) were defined as Group 1, and patients with an NPS score of 3–4 (n = 41) were defined as Group 2.

**Table 1:** Calculation of Naples prognostic score (NPS)

Variables	Cut-off value	Points	NPS group
Serum albumin (mg/dL)	≥ 4	0	Group 0: 0 point
	< 4	1	Group 1: 1–2 points
TC (mg/dL)	> 180	0	Group 2: 3–4 points
	≤ 180	1	
NLR	≤ 2.96	0	
	> 2.96	1	
LMR	> 4.44	0	
	≤ 4.44	1	

TC total cholesterol, NLR neutrophil-to-lymphocyte ratio, LMR lymphocyte-to-monocyte ratio, NPS Naples prognostic score

### Statistical analysis

Normally distributed quantitative data were expressed as mean ± standard deviation (x̄ ± SD), and one-way analysis of variance (ANOVA) was used for intergroup comparisons. Non-normally distributed quantitative data were expressed as medians and quartiles [M (P25, P75)], and a rank-sum test was used for comparison among groups. Categorical variables were described using the number of cases (%), and Chi-square test or Fisher’s exact probability test was used for intergroup comparison. Receiver operating characteristic (ROC) curves were used to analyze the predictive performance of NPSs for APE 30-day mortality. Kaplan–Meier survival analysis was used to compare the differences in 30-day mortality between different NPS subgroups, and a log-rank test was used for statistical testing of differences in survival curves. Univariate and multifactorial Cox proportional hazard models were used to analyze the association between NPS, NPS grouping, and 30-day prognosis at onset. Four models were constructed for this analysis, with each corrected for the corresponding covariates (model 1: no correction; model 2: corrected for age and sex; model 3: corrected model 2 + BMI, systolic blood pressure, pulmonary artery systolic blood pressure, heart failure, deep vein thrombosis, malignancy, and right ventricular dilatation; model 4: corrected model 3 + D-dimer, PO_2_, and N-terminal pro-brain natriuretic peptide [NT-proBNP]), a trend test was performed to determine the presence of a trend in NPS and 30-day risk of death from APE, and the results were visualized by drawing forest plots using the ggplot2 package. The performance of the NPS for the prediction versus observation of mortality was evaluated by calibration curves.

Statistical analyses were performed using R language statistical software (version 4.2.2). ROC curves were plotted using the “pROC” package, the “ggprism” package, the “ggplot2” package, and the “survminer” package for Kaplan–Meier survival curve analysis. All tests were two-sided, and differences with P < 0.05 were considered statistically significant.

## Results

### Baseline demographical and clinical characteristics

The association between baseline characteristics and NPS in the study patients is summarized in Table 2. Among 325 patients with APE, 157 (48.3%) were men and 168 (51.7%) were women, with a mean age of 64 ± 15 years. In our study population, 31 patients (9.54%) died within 30 days. According to the NPS system, 131 (40.3%) patients were included in Group 0 (NPS = 0), 153 (47.1%) in Group 1 (NPS = 1–2), and 113 (12.6%) in Group 2 (NPS = 3–4). There were significant differences in the following variables among the NPS groups: age (P = 0.001), heart rate (P < 0.001), systolic blood pressure (P = 0.032), heart failure (P = 0.035), malignant tumor (P = 0.016), DVT history (P = 0.032), right ventricle dilatation (P = 0.001), Alb levels (P < 0.001), total cholesterol levels (P = 0.073), NLR (P = 0.011), and LMR (P = 0.031). Compared to the APE patients in Groups 1 and 2, those in Group 3 were relatively older, had a faster heart rate, lower systolic blood pressure, lower Alb and total cholesterol levels, higher NLP, lower LMR, and comorbidities such as right heart dilatation, heart failure, or a history of DVT. However, no significant intergroup differences were noted in terms of male sex, smoking, BMI, respiratory rate, chronic lung disease, diabetes mellitus, hypertension, cerebrovascular disease, localization of thrombosis in PCTA, LVEF, PASP, admission blood gas analysis, D-dimer levels, troponin I levels, NT-proBNP, white blood cell count, hemoglobin levels, or platelet count.

**Table 2:** Baseline demographical and clinical characteristics of the study patients according to NPS.

Variables	Naples Prognostic Score group			*P*-value
	Group 0 (N = 131)	Group 1 (N = 153)	Group 2 (N = 41)	
Age, years	65.00 [56.00;69.50]	67.00 [55.00;72.00]	73.00 [65.00;79.00]	0.001
Male gender	63 (48.09%)	71 (46.41%)	23 (56.10%)	0.543
Smoking	62 (47.33%)	78 (50.98%)	17 (41.46%)	0.533
BMI, kg/m2	25.80 [20.85;31.60]	25.80 [20.50;28.90]	25.10 [23.40;27.60]	0.928
At admission				
Respiratory rate, beats/min	16.00 [15.00;21.00]	16.00 [15.00;21.00]	16.00 [15.00;18.00]	0.762
Heart rate, beats/min	84.00 [65.00;87.00]	87.00 [65.00;96.00]	99.00 [87.00;114.00]	< 0.001
Systolic blood pressure, mmHg	143.00 [127.00;152.00]	143.00 [127.00;151.00]	137.00 [114.00;152.00]	0.032
Co-morbidities				
Chronic lung disease	18 (13.74%)	22 (14.38%)	2 (4.88%)	0.256
Diabetes mellitus	8 (6.11%)	12 (7.84%)	7 (17.07%)	0.103
Hypertension	6 (4.58%)	19 (12.42%)	4 (9.76%)	0.063
Heart failure	3 (2.29%)	5 (3.27%)	5 (12.20%)	0.035
Cerebrovascular disease	13 (9.92%)	10 (6.54%)	1 (2.44%)	0.272
Malignant tumor	32 (24.43%)	20 (13.07%)	4 (9.76%)	0.016
DVT history	6 (4.58%)	10 (6.54%)	7 (17.07%)	0.032
Localization of thrombosis in PCTA				
Main pulmonary artery	29 (22.14%)	29 (18.95%)	4 (9.76%)	0.212
Lobar pulmonary artery	42 (32.06%)	31 (20.26%)	8 (19.51%)	0.05
Segmental pulmonary artery	76 (58.02%)	93 (60.78%)	29 (70.73%)	0.346
Subsegmental pulmonary artery	47 (35.88%)	41(26.80%)	15(36.59%)	0.319
Echocardiography parameters				
Right ventricle dilatation	3 (2.29%)	7 (4.58%)	8 (19.51%)	0.001
LVEF, %	59.00 [54.00;65.00]	61.00 [55.00;63.00]	57.00 [55.00;62.00]	0.644
PASP, mmHg	48.00 [44.00;56.00]	48.00 [44.00;56.00]	47.00 [44.00;58.00]	0.884
Admission blood gas analysis				
PH	7.38 [7.35;7.43]	7.38 [7.35;7.43]	7.39 [7.35;7.54]	0.101
PO_2_ ,mmHg	76.00 [76.00;86.00]	76.00 [76.00;87.00]	77.00 [74.00;86.00]	0.871
HCO_3_, mmol/L	25.00 [21.00;26.00]	24.00 [21.00;26.00]	25.00 [22.00;26.00]	0.983
Lactate, mmol/L	3.10 [1.90;3.30]	3.10 [1.90;3.60]	2.80 [1.90;3.10]	0.164
Laboratory parameters				
D-Dimer, ng/mL	3213.00 [1345.00;5432.00]	2356.00 [1234.00;5412.00]	1889.00 [1284.00;3503.00]	0.165
Troponin I, ng/mL	0.13 [0.08;0.17]	0.12 [0.06;0.17]	0.17 [0.08;0.21]	0.205
BNP, pg/mL	356.00 [124.00;453.00]	356.00 [124.00;453.00]	356.00 [124.00;678.00]	0.339
White blood cell count,×10^9^/L	9.40 [7.25;10.20]	9.40 [7.90;10.20]	9.40 [8.40;10.20]	0.934
Haemoglobin ,g/L	123.00 [111.00;146.00]	121.00 [106.00;143.00]	123.00 [109.00;143.00]	0.456
Platelet count,×10^9^/L	213.00 [134.00;256.00]	218.00 [167.00;312.00]	167.00 [134.00;256.00]	0.083
Albumin, mg/dL	3.95 [3.40;4.44]	3.79 [3.15;4.91]	3.13 [2.54;4.10]	< 0.001
Total cholesterol,mg/ dl	189.00 [184.00;194.00]	182.00 [185.00;194.00]	173.00 [165.00;179.00]	0.073
NLR	3.36 [2.21;4.06]	4.11 [2.49;4.46]	5.56 [4.11;8.99]	0.011
LMR	4.65 [4.59;4.87]	4.65 [4.02;4.87]	3.69 [2.46;3.99]	0.031

Abbreviations: DVT, deep vein thrombosis; HCO3, bicarbonate; PCTA, pulmonary CT angiography; LVEF, left ventricular ejection fraction; PASP, pulmonary artery systolic pressure; BNP,brain-type natriuretic peptide; NLR ,neutrophil to lymphocyte ratio ; LMR, lymphocyte to monocyte ratio

### Prognostic value of NPS

**Fig. 2:**
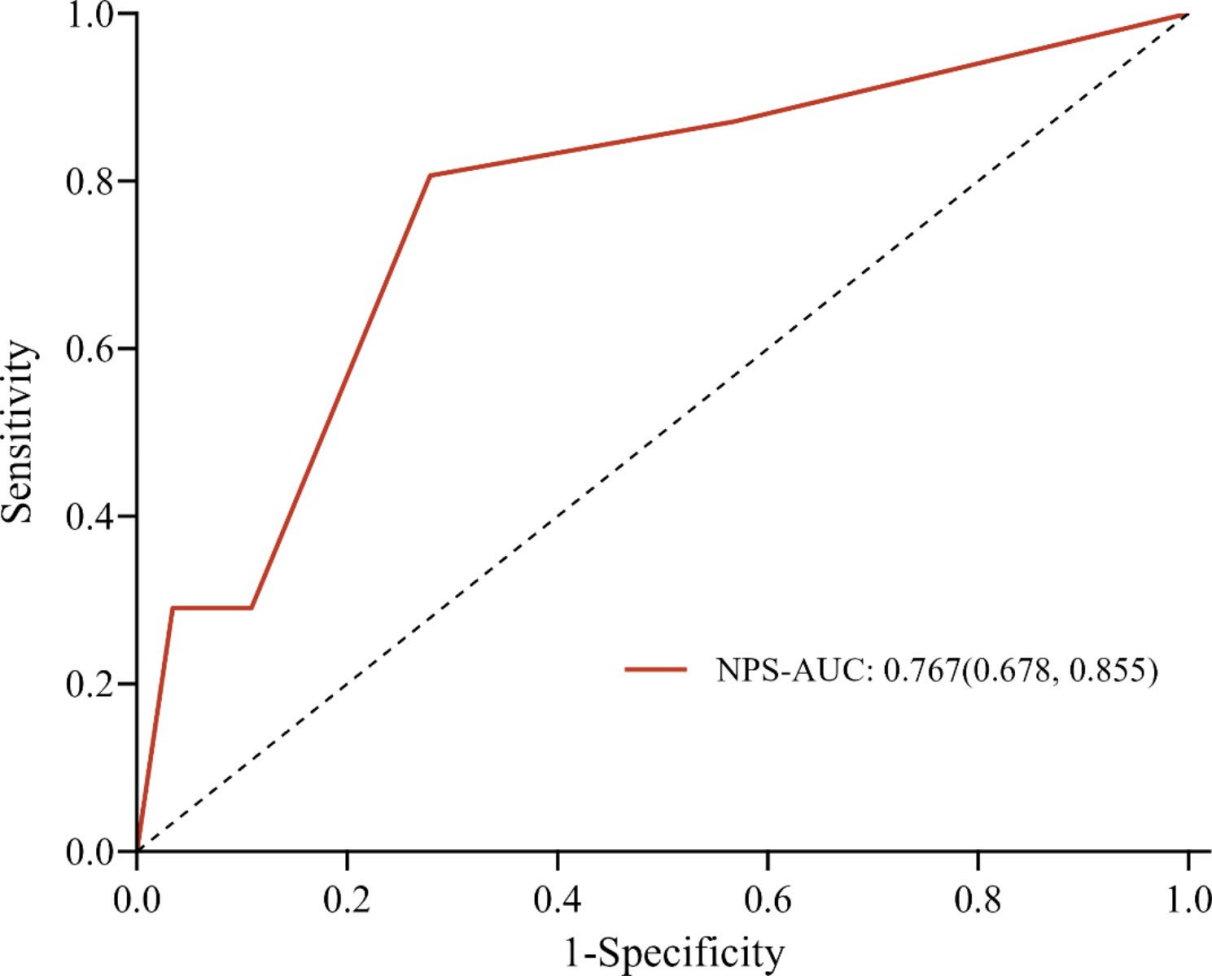
Receiver operating characteristic curves showing the predictive value of Naples Prognostic Scores for predicting all-cause mortality within 30 days in patients with acute pulmonary embolism

The area under the ROC curve (AUC) of NPS for predicting 30-day all-cause mortality in patients with APE was 0.780 (95% confidence interval [CI] = 0.678–0.855), with a sensitivity of 80.6% (95% CI = 0.667–0.946), a specificity of 72.1% (95% CI = 0.670–0.772), an accuracy of 72.9% (95% CI = 0.728–0.730), and a cut-off value of 2. The results showed that NPS had a good predictive value for 30-day all-cause mortality in patients with APE (Fig. 2).

### Survival analysis based on NPS

**Fig. 3:**
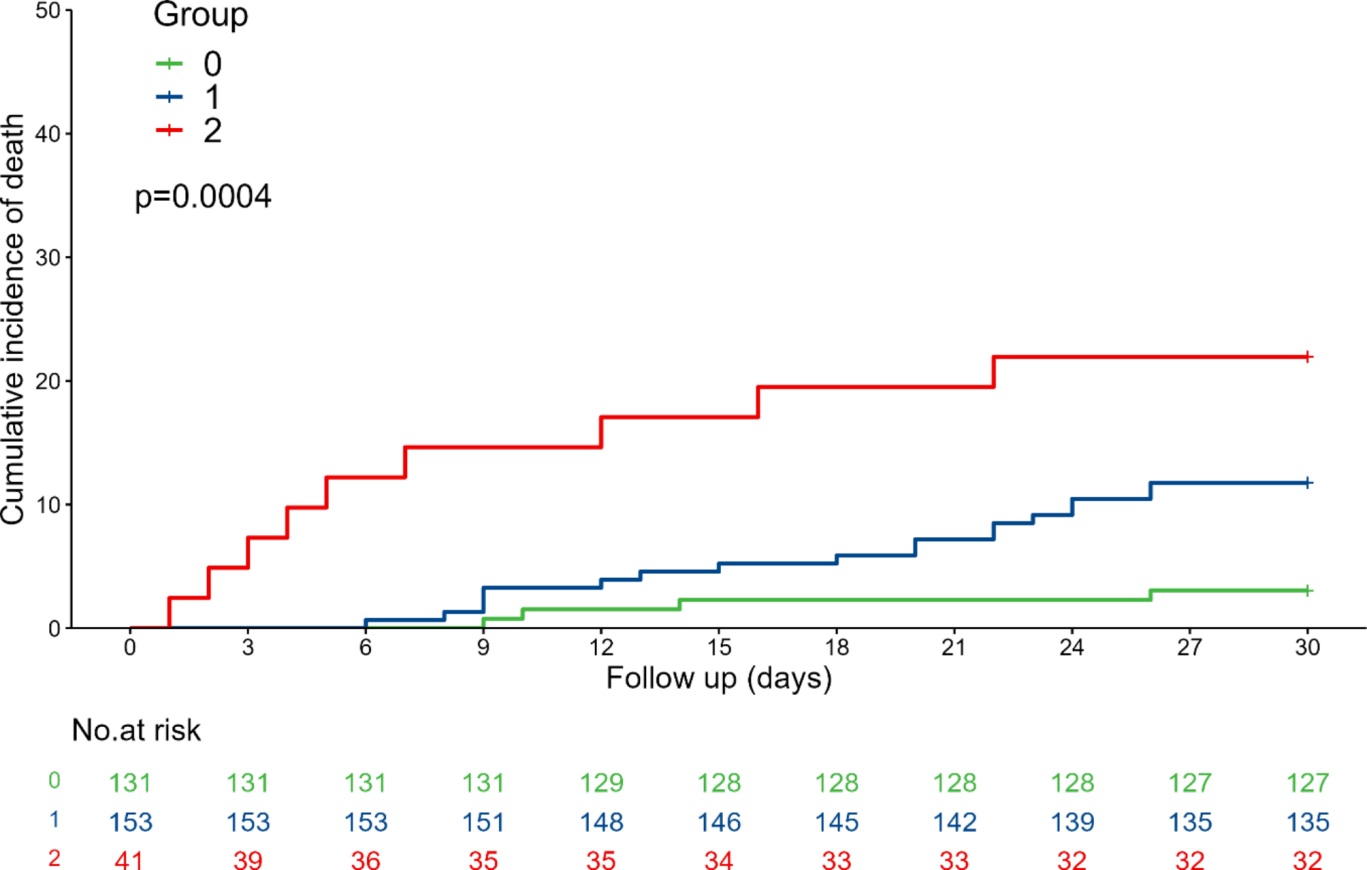
KM curves showing cumulative mortality over 30 days for different Naples Prognostic Score (NPS) subgroups in patients with acute pulmonary embolism. The difference in cumulative mortality between the different NPS subgroups was statistically significant (P = 0.0004)

**Fig. 4:**
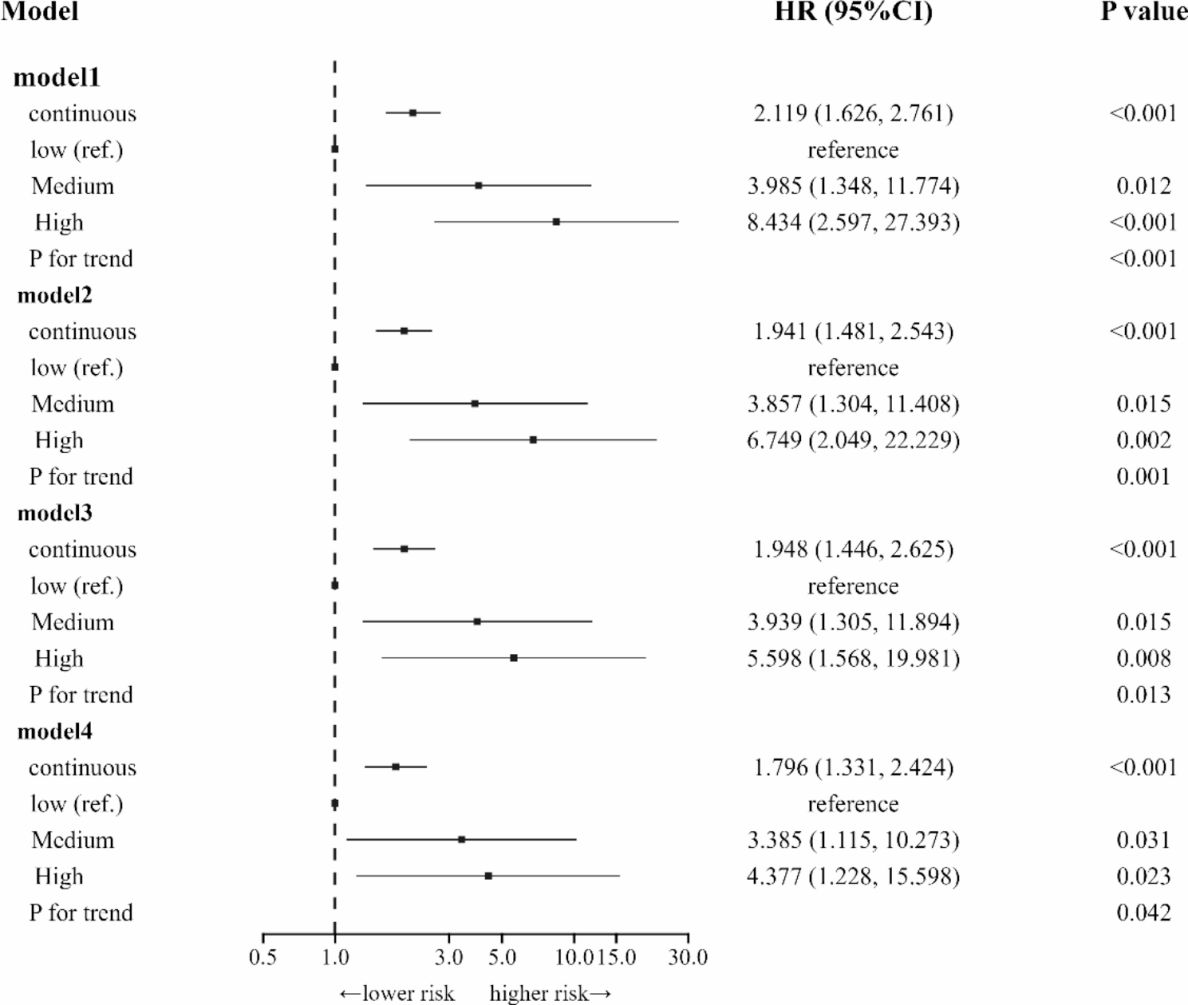
Forest plot visualizing the results of the Cox proportional risk model, which shows the relationship between continuous Naples Prognostic Scores (NPSs) and different subgroups of NPS and the risk of death within 30 days in patients with acute pulmonary embolism

**Fig. 5:**
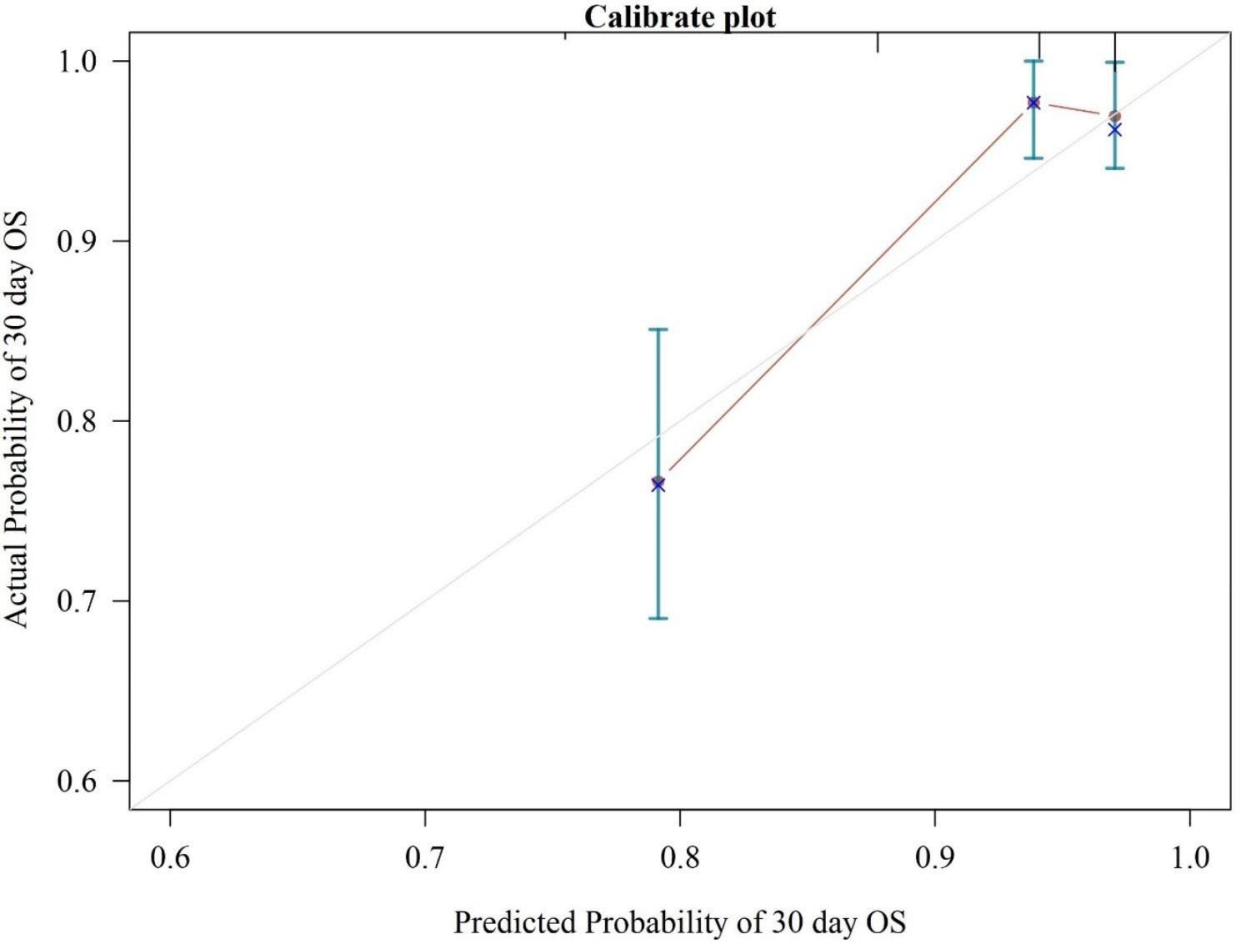
Calibration curves for the Naples Prognostic Scores (NPSs) predicted probability of 30 days survival. The dotted line represents the ideal curve where the predicted value is the same as the observed value. X-axis: survival as predicted by the NPS; Y-axis: actual survival in the cohort

In the present study, Kaplan-Meier (KM) curves were used to indicate 30-day cumulative mortality in patients with APE in different NPS subgroups (Fig. 3). The results showed that the patients with APE in Group 2 had the highest risk of all-cause mortality compared with the other two groups based on NPS grouping (log-rank test, P = 0.0004). A forest plot visualizing the results of the Cox proportional hazard model showed the relationship between continuous NPSs and different subgroups of NPS and the risk of death within 30 days in patients with APE (Fig. 4). Uncorrected models showed that continuous NPSs and NPS groupings were associated with all-cause mortality within 30 days in patients with APE. This relationship remained significant after adjustment for age, sex, BMI, systolic blood pressure, pulmonary artery systolic blood pressure, heart failure, deep vein thrombosis, malignancy, and right ventricular dilatation. In model 4, further correction for D-dimer, PO_2_, and NT-proBNP levels as well as the continuous NPS score was associated with an increased risk of death within 30 days in patients with APE (hazard ratio [HR] = 1.796 [1.331–2.424], P < 0.001). Compared with Group 0, the risk of all-cause death within 30 days in patients with APE in Groups 1 and 2 was significantly increased by 239% (HR = 3.385 [1.115–10.273], P = 0.031) and 338% (HR = 4.377 [1.228–15.598], P = 0.023), respectively, and the trend test showed statistical differences (P = 0.042). Subsequently, we evaluated the preliminary consistency between the predicted mortality and the observed mortality by generating a calibration curve (Fig. 5).

## Discussion

The clinical manifestations of APE range from asymptomatic to hemodynamic disturbances and even death. Studies have shown that the 7-day all-cause mortality rate for APE was 1.9–2.9% and that the 30-day all-cause mortality rate for APE was 4.9–23.8% [11, 12]. The present study showed a 30-day mortality rate of 9.54% (31/325), which was generally consistent with previous studies. In addition, this study showed that NPS is an independent prognostic marker of 30-day all-cause mortality in patients with APE and that patients in NPS Groups 1 and 2 had a worse prognosis compared with NPS Group 0.

NPS not only includes serum Alb and total cholesterol levels, which reflect the nutritional status of the body, but also immunoinflammatory markers such as NLR and LMR, allowing for a more comprehensive and effective assessment of the patient’s body condition at the time of admission. Alb is closely associated with the development and progression of thrombosis [13]. Patients with lung cancer are susceptible to hypoproteinemia. When the body is in a hypoalbuminemic state, it stimulates the liver to synthesize Alb. The synthesis of coagulation factors will concurrently increase, resulting in a hypercoagulable state [13]. However, when hypoproteinemia is present, water accumulates in the interstitial spaces, resulting in increased blood viscosity and a higher risk of thrombosis [15]. In the present study, a significant intergroup difference (P < 0.001) was observed, and patients with lower Alb levels had a poorer prognosis. In addition, lipids, especially HDL cholesterol, play a key role in the metabolism of normal lung tissue [14], and some studies have shown that cholesterol is crucial in the inflammatory response after acute lung injury [15]. The pathogenesis of APE is often accompanied by an inflammatory response, which can affect the synthesis of cholesterol and the absorption and transport of hepatic lipids [16]. A study by Karatas et al. showed that total serum cholesterol levels were strongly associated with short-term prognosis and recurrence rates in patients with APE [17]. In the ROC analysis, total cholesterol levels were compared with triglyceride, LDL cholesterol, and HDL cholesterol levels, a parameter with better discriminatory power for mortality. This negative correlation of lipid levels with mortality rates is known as the lipid paradox [18]. The results of the present study were consistent with those of previous studies in that the TC in the nonsurvivor group was significantly lower than that in the survival group (P = 0.073), and the negative correlation between TC and mortality may be attributable to the depletion of lipids in the acute inflammatory response.

Previous studies have shown that NLR, which is considered a novel inflammatory marker, better reflects systemic inflammation in patients and is closely related to the prognosis of pulmonary embolism [19, 20]. The NLR values of patients in the nonsurvivor group in the present study were significantly higher than those in the survivor group, supporting the results of previous studies. In addition, inflammation and endothelial damage play important roles in the progression of APE due to oxidative stress, reperfusion injury, and elevated reactive oxygen species in the lungs of patients with APE, and LMR has been proposed as a surrogate marker of endothelial dysfunction and inflammation in different populations as it has prognostic and predictive values [21]. To the best of our knowledge, only one study has shown LMR as a novel marker of inflammation. That study found that LMR was significantly lower in nonsurvivors after APE (P < 0.001), and LMR appears to be an independent predictor of short-term mortality in patients with APE [22]. The results of our study were similar in the three NPS groups and showed that APE patients with lower LMR values had a poorer prognosis (P = 0.031).

The components of NPS (TC, Alb, NLR, and LMR) are common clinical biomarkers in daily clinical practice that provide a comprehensive picture of a patient’s inflammatory and nutritional status. The prognostic effects of NPS on patients with APE are currently unknown. In view of this, this study analyzed the effect of NPS on 30-day all-cause mortality in 325 patients with APE at admission, and the results of the comparison revealed that patients with older age, faster heart rate, lower systolic blood pressure, low Alb and total cholesterol levels, high NLP, low LMR, right heart dilatation, heart failure, malignancy, and lower extremity venous thrombosis have higher 30-day all-cause mortality, and these differences were statistically significant (P < 0.05). In addition, the results revealed that the AUC for NPS to predict all-cause death within 30 days in patients with APE was 0.780 (95% CI = 0.678–0.855), with sensitivity being 80.6% (95% CI = 0.667–0.946) and specificity being 72.1% (95% CI = 0.670–0.772). This indicated that NPS at admission has good guidance for the short-term prognosis of patients with APE. In addition, Cox multivariate analysis showed that NPS was an independent risk factor for 30-day all-cause mortality in patients with APE (P = 0.0004), and stratified analysis suggested that lower scores had a better prognosis in both uncorrected and corrected covariates models. This suggested that timely nutritional support and appropriate improvement of the inflammation and immune status during the treatment of APE patients with high NPS would improve the long-term prognosis of these patients.

The accuracy and generalisability of the PESI are now supported by derivation and validation of data from multiple countries [23, 24]. The PESI reliably and accurately identifies patients at low risk of death when assessed between 7 and 90 days of follow-up. However, the PESI uses 11 clinical variables with different assigned weights, and its scores depend on calculations that may be difficult to apply clinically. The NPS prediction rules reduce this complexity. In addition, some components of the PESI, such as a patient’s disease history and clinical presentation, may be influenced by a physician’s subjective judgement. This may lead to slightly different PESI scores for the same patient by different physicians. All components of the NPS are derived from objective laboratory tests, and the results are not affected by patient recall bias and subjective judgement of clinicians. We believe that the simplified NPS is useful because we were surprised to find that the area under the ROC curve for the predictive value of the NPS was not lower than that of the PESI [2]. Thus, the NPS, a simplified scoring system, may be more applicable to busy hospital emergency departments.

The present study has some limitations. First, potential selection bias is inevitable as this was a retrospective cohort study. Second, this study is a single-center study that lacked external validation, and the moderate sample size may be the reason for the decreased power. Therefore, a multicenter, large-scale, prospective validation study is needed. Third, we did not stratify the study patients by risk, and further studies are needed to simultaneously risk-stratify patients to validate the predictive value of this prediction tool for the prognosis of patients in different strata, with a focus on short-term all-cause mortality risk. Fourth, postdischarge NPS has not been used as a follow-up marker for patients with APE, and there is a need to prospectively validate the dynamic prognostic role of NPS in order to apply a simple and easily accessible NPS in the future to enhance risk stratification and condition assessment of patients with pulmonary embolism and to guide the clinical management of APE. Fifth, due to limitations in research design and available resources, we were unable to collect hemodynamic data in this study. Hemodynamic data could provide valuable insights into the severity and prognosis of acute pulmonary embolism. Future research endeavors should aim to incorporate these data to enhance the comprehensive assessment of patients with APE.

## Conclusion

In summary, NPS is a comprehensive prognostic model that includes inflammation and nutrition-related factors. Our study found that NPS was a novel, reliable, and multidimensional prognostic scoring system with favorable predictive performance for patients with APE. In view of this, early detection and improvement of the nutritional and inflammatory status of patients, especially in the affected population of NPS Groups 1 and 2, has the potential to improve patient survival.

## Data Availability

The datasets used and/or analyzed during the current study are available from the corresponding author on reasonable request.
